# From the elastica compass to the elastica catapult: an essay on the mechanics of soft robot arm

**DOI:** 10.1098/rspa.2016.0870

**Published:** 2017-02-22

**Authors:** C. Armanini, F. Dal Corso, D. Misseroni, D. Bigoni

**Affiliations:** DICAM, University of Trento, via Mesiano 77, 38123 Trento, Italy

**Keywords:** elastica, snap-back instability, catapult

## Abstract

An elastic rod is clamped at one end and has a dead load attached to the other (free) end. The rod is then slowly rotated using the clamp. When the load is smaller than the buckling value, the rod describes a continuous set of quasi-static forms and its end traces a (smooth, convex and simple) closed curve, which would be a circle if the rod were rigid. The closed curve is analytically determined through the integration of the Euler’s elastica, so that for sufficiently small loads the mechanical system behaves as an ‘elastica compass’. For loads higher than that of buckling, the elastica reaches a configuration from which a snap-back instability occurs, realizing a sort of ‘elastica catapult’. The whole quasi-static evolution leading to the critical configuration for snapping is calculated through the elastica and the subsequent dynamic motion simulated using two numerical procedures, one *ad hoc* developed and another based on a finite-element scheme. The theoretical results are then validated on a specially designed and built apparatus. An obvious application of the present model would be in the development of soft robotic limbs, but the results are also of interest for the optimization analysis in pole vaulting.

## 1. Introduction

The design of innovative devices for advanced applications is being driven by the need for compliant mechanisms, which are usually inspired by nature [1,2] and is part of a transition from traditional robotics to soft robotics [3–5]. Compliant mechanisms require the development and use of nonlinear mechanical models such as the Kirchhoff rod [6] and Euler’s elastica, which allow the description of large deflections in elastic bars and the modelling of snake locomotion [7,8], as well as object manipulation [9–11], useful in robotic assistance during surgery [12,13] and for physical rehabilitation [14].

**Figure 1.**
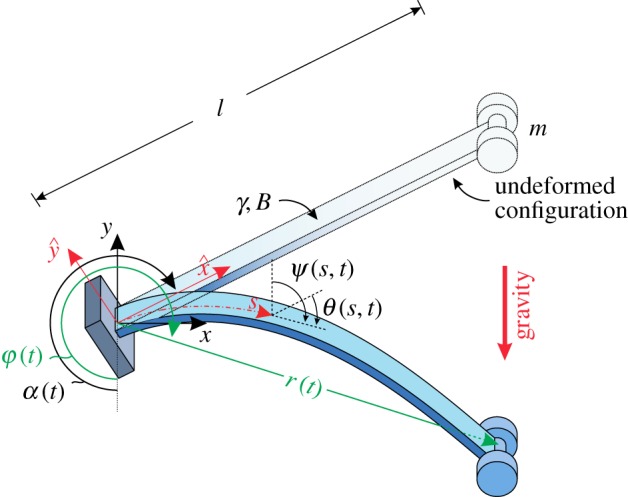
An elastic rod of length *l*, with bending stiffness *B* and linear mass density *γ*, has attached a lumped mass *m* at one end and is constrained at the other end by a slowly rotating clamp, inclined at an angle *α* (increasing function of time *t*) with respect to the direction of the gravity, so that a force *P*=*mg* is applied to an end of the rod. The rotation of the rod’s axis with respect to the undeformed (straight) configuration is measured through the angle *θ*(*s*,*t*), with *s* being the curvilinear coordinate, *s*∈[0,*l*]. The $\hat{x}-\hat{y}$ and *x*−*y* reference systems are reported, both centred at the clamp point, the former attached to the rotating clamp, whereas the latter fixed as the loading direction. The polar coordinates *r*−*φ* defining the rod’s end position are also reported. (Online version in colour.)

In this article, a basic mechanical model for a soft robot arm is addressed through new theoretical, numerical and experimental developments. In particular, the deformable mechanical system sketched in figure 1 is considered, in which an elastic rod is clamped at one end and subject to a dead load at the other. The load is provided by the weight of a mass predominantly higher than that of the rod. The clamp rotates slowly, so that starting from a configuration in which the rod is subject to purely tensile axial load, the system quasi-statically evolves in a number of elastic forms at varying clamp angle. When the load is inferior to that corresponding to buckling of the straight and uniformly compressed configuration, a whole quasi-static 360^°^ rotation of the clamp is possible and the edge of the rod describes a (smooth, convex and simple) closed curve, which is theoretically solved using Euler’s elastica. In the case of a rigid rod, this curve would be a circle, so that the mechanical system behaves as an ‘elastica compass’, thus tracing the curve described by the elastica. However, when the load is higher than that which would lead to buckling for the straight rod, an unstable configuration is quasi-statically reached, at which the rod suffers a snap-back instability and dynamically approaches another configuration, so that the system behaves as an ‘elastica catapult’.^1^ The description of the quasi-static path of the system and the determination of the unstable configuration is solved in an analytical form by means of elliptic functions through an extension of results pioneered by Wang [15], who employed a numerical integration procedure and provided asymptotic estimates valid in some particular cases (very stiff and very compliant rods, and nearly vertical equilibrium configurations).

In addition to the quasi-static solution, the dynamics of the snap instability is addressed numerically. The set-up of a numerical technique is a complex problem, which was analysed from several points of view, but not still completely solved [16–20,21]. To this purpose, two approaches are presented, one is a standard use of a finite-element software (Abaqus), whereas the other is developed as a perfection of a technique introduced for pneumatic soft robot arms [22]. The latter approach, in which the elastic rod is reduced to a nonlinear spring governed by the elastica, is elegant, but the kinematics is limited to the first deformation mode and an axial deformation and viscous damping have to be added to prevent spurious numerical instabilities, issues which may be circumvented through the finite-element approach.

Finally, the experimental validation of the elastic system was performed using a mechanical set-up specifically designed and realized at the ‘Instabilities Lab’ of the University of Trento (http://www.ing.unitn.it/dims/ssmg/). Experimental results (also available as a movie in the electronic supplementing material) fully validate the theoretical modelling, thus confirming that the elastica allows for solutions useful in the kinematics of a soft robot arm. The performance of the robot arm is also assessed in terms of (i) the maximum and minimum distances that can be reached without encountering loss of stability of the configuration and (ii) the maximum energy release that can be achieved when the system behaves as a catapult. These results open the way to a rational design of deformable robot arms and, as a side development, may find also application in the analysis of the pole vault dynamics and the optimization of athletes’ performance [23,24].

## 2. Formulation

An inextensible planar rod with bending stiffness *B*, length *l*, linear mass density *γ*, and straight in its undeformed configuration, has a lumped mass *m* attached at one end, whereas the other end is constrained by a clamp having inclination *α* with respect to gravity direction (figure 1). Denoting with *g* the gravitational acceleration, the rod is then loaded by the weight *P* owing to the lumped mass *m*, so that *P*=*mg*, and the rod distributed weight *γg* (the latter neglected in the quasi-static analysis). The clamp angle *α* smoothly and slowly increases in time *t*, so that a quasi-statically rotating clamp is realized. For simplicity of presentation, the dependence on time *t* is omitted in the notation in the following of this section. The rotation of the rod’s axis with respect to the undeformed (straight) configuration is denoted by *θ*(*s*), function of the curvilinear coordinate *s*∈[0,*l*], with *s*=0 singling out the position of the clamp (where *θ*(0)=0) and *s*=*l* of the loaded rod’s end. With respect to the undeformed straight configuration, ‘frozen’ at the inclination angle *α*, the coordinates $\hat{x}(s)$ and $\hat{y}(s)$ measure the position of the rod’s axis in the rotating system, and, owing to the inextensibility condition, are connected to the rotation field through the following differential relations [25]

2.1 $${\hat{x}}^{\prime }(s)=\cos\theta (s)\;\mathrm{and}\;{\hat{y}}^{\prime }(s)=-\sin\theta (s),$$

where a prime denotes the derivative along the curvilinear coordinate *s*. The position can be described through the coordinates *x*(*s*) and *y*(*s*) (the former orthogonal and the latter parallel, but with opposite direction, to the gravity), which are connected with the positions $\hat{x}(s)$ and $\hat{y}(s)$ through the following relationships

2.2 $$x(s)=-\hat{x}(s)\sin\alpha +\hat{y}(s)\cos\alpha ,\;y(s)=-\hat{x}(s)\cos\alpha -\hat{y}(s)\sin\alpha ,$$

so that the kinematical constraint, equation (2.1), implies

2.3 $$x^{\prime }(s)=-\sin[\theta (s)+\alpha ]\;\mathrm{and}\;y^{\prime }(s)=-\cos[\theta (s)+\alpha ].$$

During the rotation, the position of the clamp (*s*=0) is considered fixed and taken as the origin of the reference systems,

2.4 $$x(0)=y(0)=\hat{x}(0)=\hat{y}(0)=0.$$

Neglecting the rotational inertia of the lumped mass and of the rod, the Lagrangian functional for the system is given by

2.5 $$L=T-V-\int_{0}^{l}N_{x}(s)\{x^{\prime }(s)+\sin[\theta (s)+\alpha ]\}\;\text{d}s-\int_{0}^{l}N_{y}(s)\{y^{\prime }(s)+\cos[\theta (s)+\alpha ]\}\;\text{d}s,$$

where *N*_*x*_(*s*) and *N*_*y*_(*s*) are the internal forces aligned in parallel with the *x*- and *y*-directions (which play the role of Lagrangian multipliers), T is the kinetic energy of the system

2.6 $$T=\frac{1}{2}m[{\dot{x}}^{2}(l)+{\dot{y}}^{2}(l)]+\frac{1}{2}\int_{0}^{l}\gamma [{\dot{x}}^{2}(s)+{\dot{y}}^{2}(s)]\;ds,$$

(with a dot denoting the time derivative) and V is the sum of the elastic energy stored within the rod and the negative of the work done by the dead load *P* and that by the rod distributed weight *γg*,

2.7 $$V=\frac{1}{2}\int_{0}^{l}B\theta^{\prime }{\left(s\right)}^{2}\;ds+Py(l)+\int_{0}^{l}\gamma gy(s)\;\text{d}s.$$

The governing equations can be derived through application of the least action principle on the functional

2.8 $$A=\int_{t_{0}}^{t^{*}}L\;\text{d}t,$$

with *t*_0_ and t* being arbitrary initial and final instants of the analysed time interval. Reference is made to a perturbation in the fields {*x*(*s*),*y*(*s*),*θ*(*s*)} by means of the small parameter *ϵ* and of the field variations {*x*_var_(*s*), *y*_var_(*s*), *θ*_var_(*s*)} satisfying the time and space conditions

2.9 $$\begin{array}{rl} x_{\mathrm{var}}(s) & =y_{\mathrm{var}}(s)=\theta_{\mathrm{var}}(s)=0,\;\;\text{for }\;t=t_{0}\;\text{and}\;t=t^{*}\\ \;\text{and}\;x_{\mathrm{var}}(0) & =y_{\mathrm{var}}(0)=\theta_{\mathrm{var}}(0)=0,\;t\in [t_{0},t^{*}]. \end{array}\}$$

Therefore, the minimum of the functional A, equation (2.8), is obtained by imposing the following condition

2.10 $$\begin{array}{rl} & \int_{0}^{l}[\gamma \ddot{x}(s)-N_{x}^{\prime }(s)]x_{\mathrm{var}}(s)\;\text{d}s+\int_{0}^{l}\{\gamma [g+\ddot{y}(s)]-N_{y}^{\prime }(s)\}y_{\mathrm{var}}(s)\;\text{d}s\\ & \;+\int_{0}^{l}\{B\theta^{\prime \prime }(s)-N_{x}(s)\cos[\theta (s)+\alpha ]+N_{y}(s)\sin[\theta (s)+\alpha ]\}\theta_{\mathrm{var}}(s)\;\text{d}s\\ & \;+[m\ddot{x}(l)+N_{x}(l)]x_{\mathrm{var}}(l)+[m\ddot{y}(l)+P+N_{y}(l)]y_{\mathrm{var}}(l)-B\theta^{\prime }(l)\theta_{\mathrm{var}}(l)=0, \end{array}$$

providing finally the expressions

2.11 $$\begin{array}{rl} & B\theta^{\prime \prime }(s)-N_{x}(s)\cos[\theta (s)+\alpha ]+N_{y}(s)\sin[\theta (s)+\alpha ]=0,\\ & N_{x}^{\prime }(s)=\gamma \ddot{x}(s)\\ \;\text{and}\; & N_{y}^{\prime }(s)=\gamma [g+\ddot{y}(s)], \end{array}\}$$

and the boundary conditions

2.12 $$\begin{array}{rl} \theta^{\prime }(l) & =0,\\ N_{x}(l) & =-m\ddot{x}(l)\\ \;\text{and}\;N_{y}(l) & =-m[g+\ddot{y}(l)]. \end{array}\}$$

## 3. Quasi-static response

The weight of the rod *γgl* is considered here negligible when compared with that of the lumped mass attached at the end of the rod *P*. Moreover, when quasi-static conditions prevail, the acceleration of the rod’s axis can be neglected, $\ddot{x}(s)=\ddot{y}(s)=0$, so that from the boundary conditions (2.12)_2_ and (2.12)_3_, and the differential equations (2.11)_2_ and (2.11)_3_, the rod’s internal forces result to be constant along the rod

3.1 $$N_{x}(s)=0\;\mathrm{and}\;N_{y}(s)=-P,$$

so that the governing equation for the rotation field (2.11)_1_ reduces to the *elastica* [25,26]

3.2 $$B\theta^{\prime \prime }(s)-P\sin[\theta (s)+\alpha ]=0,$$

to be complemented with the boundary conditions, equation (2.12)_1_ and *θ*(0)=0.

Introducing the symbol λ^2^=*P*/*B* and the auxiliary rotation *ψ*(*s*)=*θ*(*s*)+*α*−*π* (measuring the inclination of the rod tangent with respect to the *y* axis, see figure 1), the elastica (3.2) and the boundary conditions can be rewritten as

3.3 $$\psi^{\prime \prime }(s)+\lambda^{2}\sin\psi (s)=0,\;\psi (0)=\alpha -\pi \;\mathrm{and}\;\psi^{\prime }(l)=0,$$

so that, through the following change of variables

3.4 $$k^{2}=\sin^{2}\left(\frac{\psi (l)}{2}\right),\;k^{2}\sin^{2}\sigma (s)=\sin^{2}\left(\frac{\psi (s)}{2}\right)\;\mathrm{and}\;\sigma_{0}=\arcsin\left[\frac{1}{k}\sin\left(\frac{\alpha -\pi }{2}\right)\right],$$

the integration of the differential problem (3.3) leads to the following expression providing the relation between the applied load *P* and the rotation *θ*(*l*)=*θ*_*l*_ at the loaded end of the rod

3.5 $$P=\frac{B}{l^{2}}{\left[(2n-1)K(k)-K(\sigma_{0},k)\right]}^{2}.$$

In equation (3.5), *n*=1,2,3… is an integer representing the *n*th mode solution, whereas K(k) and $K(\sigma_{0},k)$ are, respectively, the complete and the incomplete elliptic integral of the first kind of modulus *k*

3.6 $$K(a,k)=\int_{0}^{a}\frac{d\sigma }{\sqrt{1-k^{2}\sin^{2}\sigma }}\;\mathrm{and}\;\;K(k)=K\left(\frac{\pi }{2},k\right).$$

In the following, the analysis is mainly addressed to the deformation mode *n*=1, because only the equilibrium configurations related to this mode are stable [25,27]. However (when existing), the unstable equilibrium configurations related to deformation mode with *n*≠1 will also be considered in the case *P*/*P*_cr_>4 to provide the whole equilibrium paths of the system.

Once the rotation *θ*_*l*_ is computed from the nonlinear equation (3.5) for a given load *P* and a clamp angle *α*, the rotation field *θ*(*s*) is obtained from integration of equation (3.3) as the solution of the equation

3.7 $$\cos^{2}\left(\frac{\theta (s)+\alpha }{2}\right)=k^{2}\;{\text{Sn}}^{2}[s\lambda +K[\sigma_{0},k],k],$$

where Sn(⋅,*k*) represents the sinus of the Jacobian amplitude am(⋅,*k*) of modulus *k*.

With reference to the buckling load of the purely compressed clamped rod (*α*=*π*), namely *P*_cr_=*π*^2^*B*/(4 *l*^2^), the solution of the nonlinear equation (3.5) displays the two following different behaviours:

— when *P*≤*P*_cr_, a unique value of the rotation at the loaded end *θ*_*l*_ corresponds to a unique value of the clamp inclination *α*;
— when *P*>*P*_cr_, more than one solution for the rotation at the loaded end *θ*_*l*_ may exist when the clamp inclination *α* falls within the interval (2*π*−*α*_s_,*α*_s_), with *α*_s_∈[*π*,2*π*].

**Figure 2.**
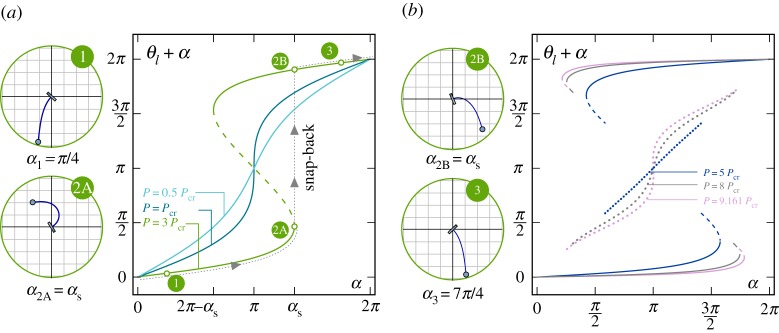
Rotation of the loaded end of the rod *θ*_*l*_+*α* versus clamp angle *α* as solution of equation (3.5), for different ratios *P*/*P*_cr_, {0.5,1,3} (*a*) and {5,8,9.161} (*b*), showing the possibility of multiple equilibrium configurations (whenever the dead load exceeds the critical load, *P*>*P*_cr_) for sets of value of the clamp inclination *α*. Stable configurations are reported as continuous lines. Unstable equilibrium configurations associated with the first (*n*=1) and second (*n*=2) mode are reported as dashed and dotted lines, respectively. Deformed equilibrium configurations for the loading condition *P*=3*P*_cr_ are reported enclosed in green circles at different clamp rotations, when only one equilibrium configuration is possible (*α*_1_=*π*/4 and *α*_3_=7*π*/4) and when two equilibrium configurations are possible (*α*_2A_=*α*_2B_=*α*_s_=1.348*π*). (Online version in colour.)

These two behaviours are highlighted in figure 2, where the rotation (with respect to the vertical direction) *θ*_*l*_+*α* of the loaded rod’s end is reported as a function of the clamp inclination *α*, solution of the nonlinear equation (3.5). Six values of the ratio *P*/*P*_cr_ have been considered, namely {0.5,1,3} and {5,8,9.161} in the left and right parts of figure 2, respectively. Uniqueness of the end rotation *θ*_*l*_ as a function of *α* is displayed only when the load *P* does not exceed the critical load *P*_cr_ (case *P*=0.5*P*_cr_), whereas more than one equilibrium configuration may be found when *P*>*P*_cr_ for some set of values for the clamp angle *α*. For instance, in the case *P*=3*P*_cr_ (figure 2, left), three equilibrium configurations (related to *n*=1) are displayed for the clamp angle *α* within the interval (2*π*−*α*_s_,*α*_s_). Note that when the equilibrium configuration is not unique, only two of the equilibrium configurations are stable (represented as continuous lines in the figure), whereas the others are unstable (represented as discontinuous lines). The limit case *P*=*P*_cr_ (figure 2, left) is also reported, for which a vertical tangent is displayed at *α*=*π*, which defines the transition between the two behaviours.

For completeness, it is observed that when *P*≤*q*^2^*P*_cr_ (q∈N), the nonlinear equation (3.5) may admit solutions for *n*th mode, with *n*<*q*. For instance, when *P*∈[9,16]*P*_cr_, a total of five equilibrium configurations may exist for the same value of *α* (figure 2, right, case *P*=9.161*P*_cr_).

The uniqueness/non-uniqueness of the quasi-static solution defines two qualitatively different mechanical responses for the analysed elastic system. Indeed, considering a monotonic increase of the clamp angle *α* from 0 to 2*π*:

— when *P*≤*P*_cr_, the rotation *θ*_*l*_ changes continuously, so that the end of the rod describes a (smooth, convex and simple) closed continuous curve. In this condition, the system behaves as an *elastica compass*;
— when *P*>*P*_cr_, the rotation *θ*_*l*_ reaches a critical value (corresponding to the snap clamp inclination *α*_s_∈[*π*,2*π*]), for which a further increase in the clamp angle necessarily yields a jump in the rotation *θ*_*l*_. Such a jump involves a release of elastic energy and a dynamic snap to another, non-adjacent configuration. In this condition, the system behaves as an *elastica catapult*.

**Figure 3.**
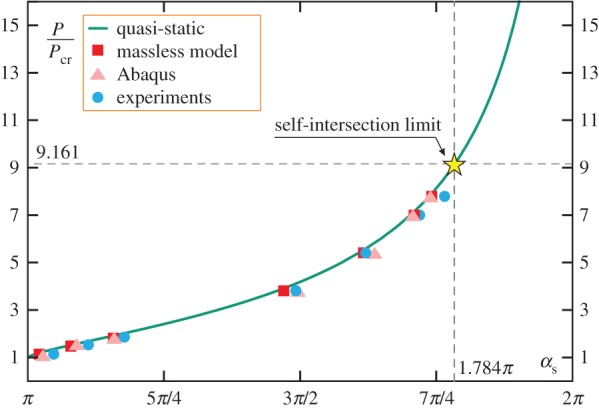
Clamp inclination *α*_s_, for which snap-back is attained, as a function of the load ratio *P*/*P*_cr_. The theoretical prediction (continuous green line) is compared to the numerical evaluation (squares and triangles) obtained from the dynamic analysis (see §7) and the experimental data (circles) measured on the developed prototype (see §8). (Online version in colour.)

The clamp angle *α*_s_ for which the snap-back instability occurs has been numerically evaluated and is reported as a function of the load ratio *P*/*P*_cr_ in figure 3. It can be noted that *α*_s_ is always greater than *π* (limit value attained when *P* coincides with the buckling load, *P*=*P*_cr_) and is an increasing function of the applied load, so that as the applied load increases the snap-back occurs ‘later’. Results from the dynamic analyses (provided in §7) and from the experimental tests on the developed physical prototype (described in §8) are also reported in the figure. The snap condition related to the load value *P*_*si*_, defining the limit condition of self-intersection (see §4), is highlighted. The agreement of the analytical predictions with the results obtained from both the experimental tests and the numerical simulations substantiates the assumptions of quasi-static motion before snap and rod’s inextensibility adopted in the analytical evaluations.

## 4. The elastica compass and the elastica catapult

A further insight into the mechanics of the elastica compass and catapult requires the development of the rod’s kinematics.

From the rotation field (3.7), the integration of equation (2.3) provides the position of the rod’s axis, that in the *x*−*y* reference system becomes (see [28,29])

4.1 $$x(s)=A_{1}(s)l\;\mathrm{and}\;y(s)=A_{2}(s)l,$$

where

4.2 $$\begin{array}{rl} A_{1}(s) & =-\frac{2k}{\lambda l}\{\text{Cn}(s\lambda +K(\sigma_{0},k),k)-\text{Cn}(K(\sigma_{0},k),k)\}\\ \;\text{and}\;A_{2}(s) & =-\frac{s}{l}+\frac{2}{\lambda l}\{E(\text{am}(s\lambda +K(\sigma_{0},k),k))-E(\sigma_{0},k)\}, \end{array}\}$$

and Cn denotes the Jacobi cosine amplitude function Cn(⋅,k)=cos⁡(am(⋅,k)), whereas *E* is the incomplete elliptic integral of the second kind,

4.3 $$E(a,k)=\int_{0}^{a}\sqrt{1-k^{2}\sin^{2}\sigma }\;d\sigma .$$

**Figure 4.**
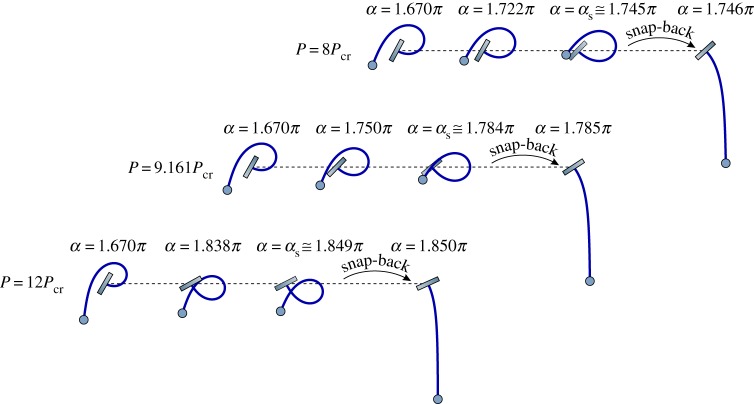
A sequence of quasi-static configurations of the elastica catapult (*P*/*P*_cr_> 1) at increasing the clamp angle *α* for different values of *P*/*P*_cr_ ending with snap in the equilibrium configuration. The self-intersection of the rod occurring for *P*≥*P*_si_ is displayed. In particular, the limit case of self-intersection is shown in the central sequence (*P*=*P*_si_≈9.161*P*_cr_), whereas self-intersection is shown in the lower sequence (*P*=12*P*_cr_). (Online version in colour.)

Considering a fixed weight *P*, the quasi-static evolution of the deformed configuration can be represented by varying the clamp angle *α* using the kinematical description (4.1) (figure 4). It is found that, when *P*=*P*_*si*_≈9.161*P*_cr_, the deformed configuration displays a self-contact point with the clamp at the verge of the snap-back, *α*_s_≈1.784*π*. Therefore, the load *P*_*si*_ defines the lowest value of the load needed to achieve self-intersection of the elastic rod during rotation of the clamp. Depending on the out-of-plane geometry of the rod, two behaviours can be attained in the case *P*>*P*_*si*_: (i) if the geometry permits self-intersection, the present solution holds and self-intersecting elastica are displayed, whereas (ii) if the geometry does not permit self-intersection, a contact point is formed within the configuration of the rod at increasing the clamp rotation. The response of the system in the case *P*>*P*_*si*_ will be the subject of future investigation.

**Figure 5.**
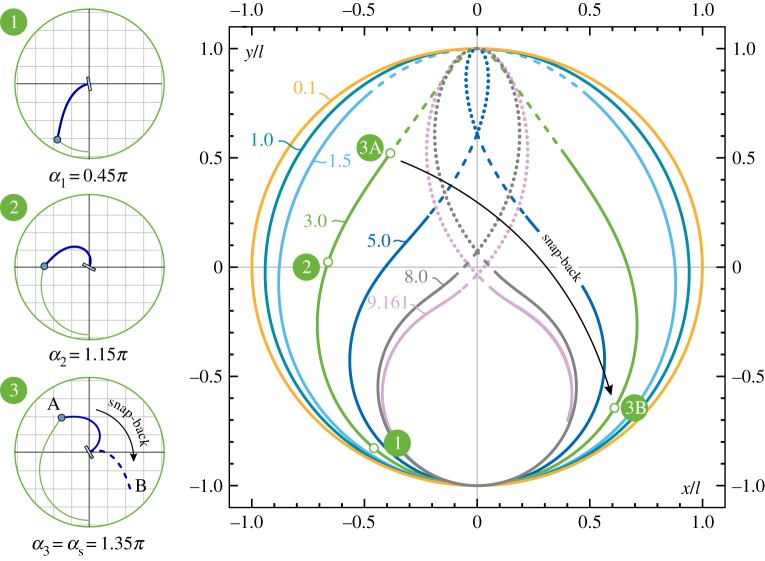
‘Pencil lead’ trajectories drawn by the elastica compass/catapult within the dimensionless plane *x*/*l*–*y*/*l* for different values of *P*/*P*_cr_. Stable configurations are reported as continuous lines. Unstable positions are marked as discontinuous lines, dashed lines for the first mode and dotted lines for the second mode. Deformed configurations of the elastic rod for specific end positions are reported in the circles on the left for the case *P*=3*P*_cr_. (Online version in colour.)

The trajectory travelled by the loaded end (playing the role of the pencil lead of the ‘elastica compass’) can be traced by evaluating the coordinates (4.1) at the loaded end, *s*=*l*, at varying clamp inclination *α*∈[0,2*π*]. The quasi-static trajectories are reported in figure 5 for different values of the ratio *P*/*P*_cr_. It can be observed that the trajectories have the shape of (smooth, convex and simple) closed curves in the case *P*<*P*_cr_. Furthermore, unstable positions for the rod’s end are reported as a discontinuous line in the case *P*>*P*_cr_ (as dashed and dotted line for the first and second mode configurations, respectively), so that the snap-back instability is initiated at the point where the continuous line ends.

The position of the loaded end can also be described in a polar reference system through the radius $r=\sqrt{x{\left(l\right)}^{2}+y{\left(l\right)}^{2}}$ and the angle φ=3π/2−arctan⁡[y(l)/x(l)], which result

4.4 $$\begin{array}{rl} r & =l\sqrt{A_{1}^{2}(l)+A_{2}^{2}(l)}\\ \;\text{and}\;\varphi & =\frac{3\pi }{2}-\arctan\left(\frac{A_{2}(l)}{A_{1}(l)}\right). \end{array}\}$$

**Figure 6.**
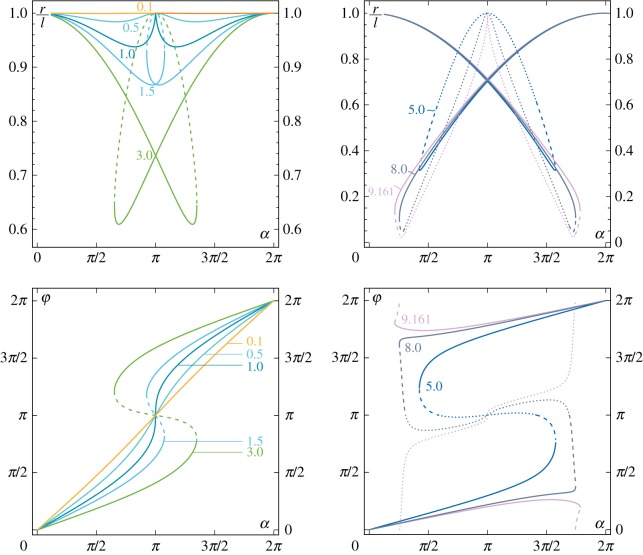
‘Pencil lead’ position of the elastica compass/catapult described in terms of the radius *r* (top) and the angle *φ* (bottom) as functions of the clamp angle *α* for different load ratios *P*/*P*_cr_, {0.1,0.5,1,1.5,3} (left part) and {5,8,9.161} (right part). Unstable configurations are reported as discontinuous lines, dashed and dotted for first and second mode configurations, respectively. (Online version in colour.)

The polar coordinates *r* (made dimensionless through division of the rod’s length *l*) and *φ* which describe the rod’s end trajectory are reported in figure 6 at varying the clamp angle *α* for different values of the ratio *P*/*P*_cr_.

From figures 5 and 6, it can be observed that

**Figure 7.**
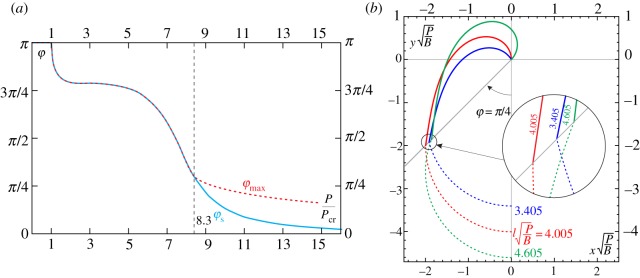
Maximum polar angle $\varphi_{\max}$ (red/dashed line) and polar angle at the verge of the snap-back instability *φ*_s_ (blue/continuous line) as functions of the load ratio *P*/*P*_cr_ (*a*). The two polar angles coincide for the range *P*/*P*_cr_≤8.3. Initial part of the trajectory (dashed lines) travelled by the loaded end of three soft robot arms reported within the dimensionless plane $x\sqrt{P/B}-y\sqrt{P/B}$. The three systems have the same bending stiffness *B*, are subject to the same weight *P*, but differ in the soft arm length, $l=\{3.405,4.005,4.605\}\sqrt{B/P}$ (*b*). The deformed configurations of the three systems are drawn (continuous line) for clamp inclinations *α*={0.826,0.997,1.239}*π*, respectively, for which the hanged load lies along the polar coordinate *φ*=*π*/4. The maximum radial distance *r*_max_ is attained with the system having the length $l=4.005\sqrt{B/P}$. (Online version in colour.)

— the behaviour of the usual, namely undeformable, compass is recovered in the limit of vanishing *P*/*P*_cr_, for which the elastic rod behaves as a rigid bar, *r*(*α*)=*l* and *φ*(*α*)=*α*;
— owing to the inextensibility assumption, the loops drawn by the elastica compass always lie inside the circle of radius *l*, therefore, *r*(*α*)≤*l*;
— for dead loads smaller than the buckling load, *P*<*P*_cr_, the loops drawn by the elastica compass are nearly circular despite the large difference between *φ* and *α*. This is due to the fact that the maximum percentage decrease in the radius length is about 6%;
— for dead loads larger than the buckling load, *P*>*P*_cr_, the polar coordinate *φ* is limited by the upper bound $\varphi_{\max}(P/P_{\mathrm{cr}})=\max_{\alpha }\varphi \;(\alpha ,P/P_{\mathrm{cr}})$, described by the dashed curve reported in figure 7*a*. Defining *φ*_s_ as the polar angle at the verge of the snap-back instability, namely *φ*_s_(*P*/*P*_cr_)=*φ*(*α*_s_(*P*/*P*_cr_),*P*/*P*_cr_) (reported as continuous curve), it is observed that $\varphi_{s}(P/P_{\mathrm{cr}})\leq \varphi_{\max}(P/P_{\mathrm{cr}})$, where the equality holds for *P*/*P*_cr_≤8.3.

In figure 7*b*, three soft robot arms with the same bending stiffness *B*, subject to the same weight *P*, but differing in the soft arm length, $l=\{3.405,4.005,4.605\}\sqrt{B/P}$ are considered. Deformed configurations (continuous line) are reported within the dimensionless plane $x\sqrt{P/B}-y\sqrt{P/B}$ at clamp inclinations *α*={0.826,0.997,1.239}*π*, for which the loaded ends of all the three systems have the same polar coordinate *φ*=*π*/4. The comparison of the radial coordinate of the loaded ends for the three cases highlights that the maximum radial distance *r*_max_ corresponds to that of the system with arm length $l=4.005\sqrt{B/P}$. This observation implies that lengthening of the arm does not always provide an increase in the attained distance. This concept is further analysed in the next section.

Finally, the quasi-static analysis is completed by the evaluation of the reaction moment at the rotating clamp *M*(0)=−*P* *x*(*l*), which can be computed through the displacement field (4.1) as

4.5 $$M(0)=-A_{1}(l)Pl.$$

**Figure 8.**
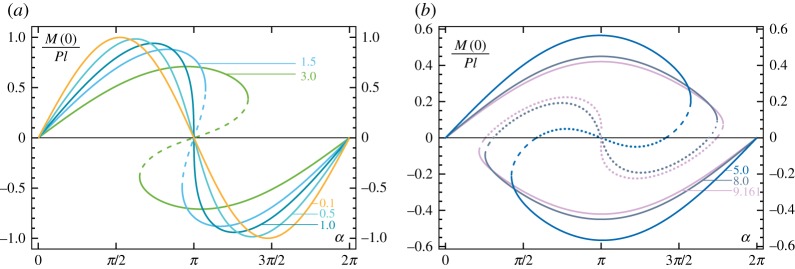
Reaction moment *M*(0) at the clamp at varying clamp angle *α* for different values of the load ratio *P*/*P*_cr_ equal to {0.1,0.5,1,1.5,3} (*a*) and to {5,8,9.161} (*b*). Moments evaluated at unstable configurations are reported as discontinuous lines, dashed and dotted for first and second mode configurations, respectively. (Online version in colour.)

The reaction moment *M*(0) is reported in figure 8, showing a change in sign at the snap-back, as a change in sign for the rod’s curvature occurs. This feature has been exploited to detect the snap inclination *α*_s_ from the results obtained with the numerical and experimental investigations explained below.

## 5. Robot’s arm performance and design

The performances of the soft robot arm are investigated in terms of extremum distances that the loaded end can attain, which represent fundamental quantities in the design of soft robot arm, to achieve targeted positions for the hanged weight.

The maximum horizontal distance *d*_*x*_, the maximum height *d*_*y*_, and the minimum radial distance *r*_min_ reached by the hanged weight during the clamp rotation and before the possible snap-back instability, are introduced as

**Figure 9.**
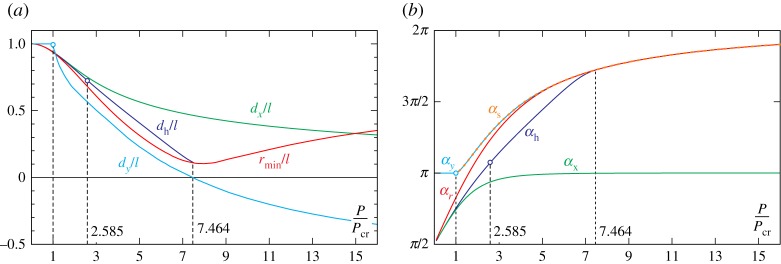
Maximum horizontal distance *d*_*x*_, maximum height *d*_*y*_, and minimum radial distance *r*_min_ (*a*) and corresponding angles *α*_*x*_, *α*_*y*_ and *α*_*r*_ (*b*) plotted as functions of the load ratio *P*/*P*_cr_. The distance *d*_h_, equation (5.2), called ‘longest horizontal reach’ [30], the related angle *α*_h_, and the snap angle curve *α*_s_ are also reported. (Online version in colour.)

5.1 $$d_{x}=\max_{\alpha }\{-x(l)\},\;d_{y}=\max_{\alpha }\{y(l)\}\;\mathrm{and}\;r_{\min}=\min_{\alpha }\{r\},$$

where *α*∈[0,*π*] for the elastica compass (*P*<*P*_cr_) and *α*∈[0,*α*_s_] for the elastica catapult (*P*>*P*_cr_). Considering fixed both the length *l* and the stiffness *B* and exploiting the kinematical description (4.1), the distances *d*_*x*_, *d*_*y*_, *r*_min_ have been evaluated at varying load *P* and are reported in figure 9*a*, together with the respective clamp rotations *α*_*x*_, *α*_*y*_ and *α*_*r*_ for which these distances are attained, figure 9 (right). The distance *d*_h_ (also called ‘longest horizontal reach’ by [30]) is defined as the horizontal distance attained by the weight when its vertical coordinate vanishes (namely the weight and the clamp are at the same height):

5.2 $$d_{h}=-x(l),\;\text{subject to the condition}\;y(l)=0,$$

and has been evaluated and reported in figure 9, together with the respective angle *α*_h_.

From figure 9, the following conclusions can be drawn at varying the load *P* and considering constant both the length *l* and the stiffness *B*.

— The maximum horizontal distance *d*_*x*_ attains its maximum value in the rigid limit (*P*/*P*_cr_=0), for which *d*_*x*_=*l*, and is a decreasing function of the load *P*. The distance *d*_*x*_ is always attained for a clamp angle *α*_*x*_∈[*π*/2,*π*].
— In the case of the elastica compass, the maximum height corresponds to the robot arm length, *d*_*y*_=*l*, independently of the load *P*≤*P*_cr_ and is attained for *α*=*π*. In the case of the elastica catapult, the maximum height *d*_*y*_ decreases at increasing load *P* and is attained at the verge of the snap-back instability, *α*_*y*_=*α*_s_. Moreover, when *P*/*P*_cr_>7.464, the weight always remains below the clamp before the occurrence of the snap-instability, *d*_*y*_<0. For such a load range, the longest reach *d*_h_ corresponds only to unstable configurations (and therefore cannot be attained).
— The minimum radial distance attains its minimum value $r_{\min}=0.104\;l$ for the load *P*/*P*_cr_=7.985. Moreover, when *P*/*P*_cr_>5.665, the minimum distance is achieved for a clamp rotation at the verge of instability (*α*_*r*_=*α*_s_).
— The distance *d*_h_ is always smaller than or equal to the horizontal distance *d*_*x*_, namely *d*_h_≤*d*_*x*_, where the equality holds only in the case of vanishing load, *P*=0. Moreover, the distance *d*_h_ is defined only when *P*/*P*_cr_<7.464, because, otherwise, the configurations are unstable at null vertical coordinate, *y*(*l*)=0.

It is worth remarking that the curves reported in figure 9 are plotted in a dimensionless plane, in which both axes are affected by a change in the rod length *l*. Therefore, playing with this length would permit maximizing of the physical distances effectively reached by the loaded end when the hanged weight *P* is kept constant (as well as the bending stiffness *B*). Such maximum distances can be evaluated by seeking the load ratios *P*/*P*_cr_ for which the following function attains a maximum

5.3 $$\sqrt{\frac{P}{P_{\mathrm{cr}}}}\frac{d_{j}(P/P_{\mathrm{cr}})}{l},\;j=x,y,h.$$

The numerical implementation of this procedure leads to the following load ratios for which the soft arm displays the maximum possible distances

5.4 $${\left(\frac{P}{P_{\mathrm{cr}}}\right)}_{d_{x}^{\max}}\to \infty ,\;{\left(\frac{P}{P_{\mathrm{cr}}}\right)}_{d_{y}^{\max}}=1\;\mathrm{and}\;{\left(\frac{P}{P_{\mathrm{cr}}}\right)}_{d_{h}^{\max}}=2.585.$$

The evaluated load ratios correspond to specific arm lengths *l* to be considered

5.5 $$l=\frac{\pi }{2}\sqrt{\frac{P}{P_{\mathrm{cr}}}}\sqrt{\frac{B}{P}}$$

for achieving the maximum distances, namely

5.6 $$\frac{d_{y}}{l}=1\;\mathrm{and}\;\frac{d_{h}}{l}=0.726,$$

while the maximum distance *d*_*x*_ is represented by the trivial limit case of infinitely long arm, l→∞. The maximum distances *d*_*y*_ and *d*_h_ can be evaluated in terms of the hanged load *P* and the bending stiffness *B* as

5.7 $$\begin{array}{rl} d_{y} & =1.571\sqrt{\frac{B}{P}},\;\text{for}\;l=1.571\sqrt{\frac{B}{P}}\\ \;\text{and}\;d_{h} & =1.833\sqrt{\frac{B}{P}},\;\text{for}\;l=2.525\sqrt{\frac{B}{P}}, \end{array}\}$$

attained for the following clamp inclinations

5.8 $$\alpha_{y}=\pi \;\mathrm{and}\;\alpha_{h}=1.072\pi .$$

The maximum distances *d*_*y*_ and *d*_h_ and related clamp inclination *α*_*y*_ and *α*_h_ are reported as spots in figure 9.

The optimization of the soft robot arm in terms of kinematics is completed by considering the maximum radial distance $r_{\max}(\varphi )$, defined as the maximization of the radial distance *r* for a given polar angle *φ* (keeping fixed the applied load *P* and the bending stiffness *B*). Following equation (5.3), the load ratio *P*/*P*_cr_ for which the maximum radial distance *r*_max_ is achieved can be found by seeking the maximum of the function

5.9 $$\sqrt{\frac{P}{P_{\mathrm{cr}}}}\frac{r(P/P_{\mathrm{cr}},\varphi )}{l}.$$

**Figure 10.**
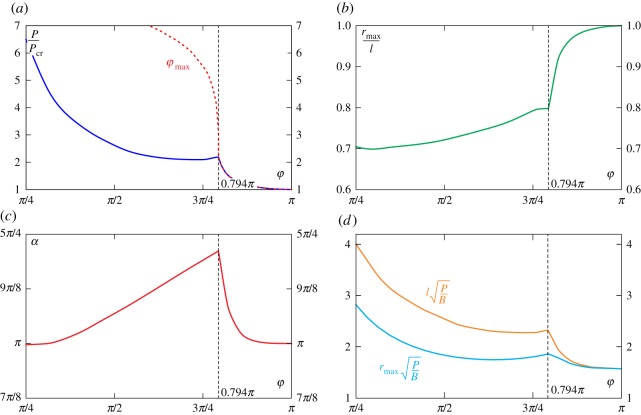
*Optimal* load ratio *P*/*P*_cr_ as a function of the polar coordinate *φ* for which equation (5.3) is maximized (*a*). Maximum radial distance *r*_max_ normalized through division by the length *l* (*b*) and corresponding clamp inclination *α* (*c*) as functions of the angle *φ*. Arm length *l* and the maximum radial distance *r*_max_ (made dimensionless through division by the fixed parameters *P* and *B*) at varying the polar angle *φ* (*d*). (Online version in colour.)

Restricting the attention to the polar coordinate *φ* ranging between *π*/4 and *π*, the *optimal* load ratio *P*/*P*_cr_ has been reported in figure 10*a* as the result of the maximization of equation (5.3). Under this loading condition, the maximum radial distance *r*_max_ (normalized through division by the length *l*) and the clamp inclination *α* (for which such a distance is attained) are evaluated at varying the angle *φ* in figure 10 (respectively, *b* and *c*). Furthermore, the arm length *l* and the maximum radial distance *r*_max_ (made dimensionless through division by the constant parameters *P* and *B*) are provided in figure 10*d*.

It can be observed that

— all the curves display a jump in their first derivative for the value *φ*=0.794*π*. For polar coordinates *φ* higher than this value, the load ratio *P*/*P*_cr_ maximizing the radial distance corresponds to an angle equal to the maximum possible for that loading condition, $\varphi =\varphi_{\max}$ (figure 10*a*);
— the maximum radial distance is never observed for loads *P* smaller than the critical one *P*_cr_;
— the results reported in figure 10*d* represent an extension of the recent result by Wang [30] and Batista [28], referred to the case of an hanged load located along the horizontal axis *x* (at *φ*=*π*/2) and expressed by the distance *d*_h_, see equation (5.7)_2_. On the other hand, in the limit of *φ*=*π*, the value of the radial distance *r*_max_ approaches the value of the length *l*, see equation (5.7)_1_.
— The curve reported in figure 10*d* defines the values of the optimal lengths *l* for which the maximum radial distance is attained. In other words, lengthening does not always realize an increase in the achieved distances so that, using words similar to Wang [30], *shortening of rods may provide longer distances*, a key concept in the design of soft robot arms.

An applicative example of kinematic performance design towards the achievement of the maximum radial distance *r*_max_, for given load *P* and stiffness *B*, is reported in figure 7*b* for three soft robot arms differing only in their length. The deformed configurations, subject to the condition of loaded end lying along the polar coordinate *φ*=*π*/4, are shown for the three systems. It is observed that the maximum distance is attained when the soft arm has the optimal length $l=4.005\sqrt{B/P}$, which value is provided by the curve reported in figure 10*d* for *φ*=*π*/4.

Finally, with reference to the strength, the design of the arm cross section is ruled by the maximum bending moment $M_{\max}$ experienced along the elastic arm, *s*∈[0,*l*], at varying the clamp inclination

$$M_{\max}=\max_{\alpha ,s\in [0,l]}M(s).$$

For the considered load range, *P*/*P*_cr_∈[0,16], it is numerically found that the maximum bending moment always occurs at the clamp (*s*=0) for the clamp inclination *α*=*α*_*x*_, so that

5.10 $$M_{\max}=Pd_{x}.$$

## 6. Energy release

In the case of the elastic catapult, when the clamp inclination approaches the value *α*_s_, the snap-back instability induces a dynamic motion in the elastic system. Such a motion is due to the energy release of the system, provided in terms of the release of both elastic energy stored in the rod and the potential energy of the hanged load.

With reference to the two stable equilibrium configurations associated to the clamp rotation *α*∈[2*π*−*α*_s_,*α*_s_], the energy difference ΔE can be computed as

6.1 $$\Delta E(P,\alpha )=\frac{B}{2}\int_{0}^{l}[[\theta^{\prime 2}(s)]]\;ds+P[[y(l)]],$$

where the symbol [[⋅]] denotes the jump of the relevant argument evaluated for the configuration related to *θ*_*l*_+*α*∈[0,*π*] and for that related to *θ*_*l*_+*α*∈[*π*,2*π*]. Considering the kinematics at equilibrium, equations (2.1), (3.4)_1_ and (4.1), the energy difference ΔE (made dimensionless through division by *B*/*l*) can be re-written as

6.2 $$\frac{\Delta E(P,\alpha )l}{B}=[[2A_{1}(l)+\cos(\theta_{l}+\alpha )]]\frac{\pi^{2}}{4}\frac{P}{P_{\mathrm{cr}}}.$$

Because of the symmetry of the paths in figure 2, the following relation for the energy difference holds

6.3 $$\Delta E(P,2\pi -\alpha )=-\Delta E(P,\alpha ),\;\alpha \in [\pi ,\alpha_{s}],$$

so that

6.4 ΔE(P,α=π)=0,

and therefore the clamp angle *α*=*π* represents the Maxwell line for the system [25,31,32].

**Figure 11.**
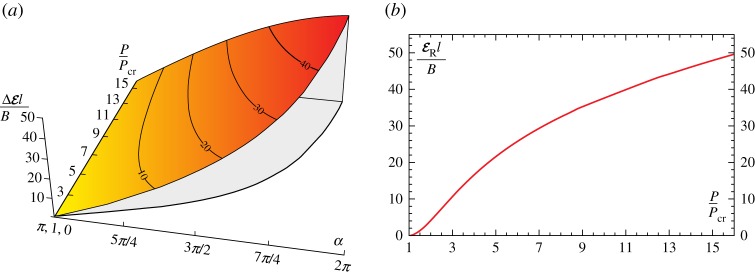
Energy difference ΔE, equation (6.1), normalized through division by *B*/*l*, as a function of the load ratio *P*/*P*_cr_ and the clamp inclination *α* (*a*). Energy release $E_{R}$ at snap-back (*α*=*α*_s_), equation (6.5), as a function of the load ratio *P*/*P*_cr_ (*b*). (Online version in colour.)

The energy difference ΔE(P,α) is reported in figure 11*a* at varying the load ratio *P*/*P*_cr_ and the clamp angle *α*∈[*π*,*α*_s_]. It is observed that the maximum energy difference, ${\text{max}}_{\alpha }\Delta E(P,\alpha )$ is attained at the verge of the snap instability, namely, at the clamp angle *α*=*α*_s_. Therefore, the energy release $E_{R}$ occurring at the snap-back, evaluated from the quasi-static solution, corresponds to the maximum energy difference possible for the elastic system,

6.5 $$E_{R}(P)=\Delta E(P,\alpha_{s})$$

which is reported as a function of the applied load *P* in figure 11*b*.

## 7. Snap-back dynamics

The dynamics of the rotating cantilever rod, occurring as a consequence of the instability when the clamp is quasi-statically rotating, is here investigated through two models: (i) a simplified model (called ‘massless rod model’) where the rod’s inertia is neglected (because considered small when compared to the inertia of the point mass *m*) and regularizations are introduced through viscous damping owing to air drag acting on the mass and axial compliance of the clamp constraint, and (ii) a finite-element model (performed in Abaqus v. 14.3), in which the inertia of the rod is fully accounted for and Rayleigh internal damping is present. A slightly different version of model (i), which will be presented below, was proposed in the modelling of a tube-type manipulator arm by Snyder & Wilson [22].

It is shown that both models provide useful insights into the dynamics of the mechanical system after snap-back instability. However, while model (ii) is capable of describing the complete dynamic evolution of the system up to a wide range of loading parameters, the model (i) can predict the full dynamics only until deformation modes higher than the first do not enter the solution (in other words, the massless rod model works correctly for *P*/*P*_cr_ smaller than ≈2).

### (a) Massless rod model

In the model where the rod’s mass *γ* (as well as any rotational inertia) is neglected, the dynamics of the whole system is characterized only by the translational inertia of the lumped mass *m* at the rod’s free end. Therefore, the rotation field of the rod’s axis is governed by

**Figure 12.**
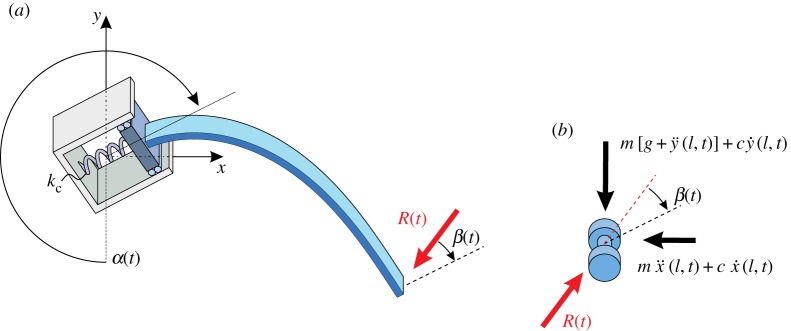
Free-body diagram of the massless rod model: schemes of the massless elastic rod (*a*) and the lumped mass (*b*). Regularization of the solution obtained with this simplified model is achieved through the introduction of the viscous coefficient *c* owing to air drag and the finite stiffness *k*_c_ of the clamp. (Online version in colour.)

7.1 $$B\theta^{\prime \prime }(s,t)-R(t)\sin[\theta (s,t)+\beta (t)]=0,$$

where the load *R*(*t*) is the force resultant acting at the free end and *β*(*t*) is the inclination angle of the force resultant with respect to the (straight) undeformed configuration (figure 12),

7.2 $$R(t)=\sqrt{R_{x}{\left(t\right)}^{2}+R_{y}{\left(t\right)}^{2}}\;\mathrm{and}\;\beta (t)=\alpha (t)+\arctan\frac{R_{x}(t)}{R_{y}(t)}.$$

In equation (7.2), *R*_*x*_(*t*) and *R*_*y*_(*t*) are the resultant components of *R*(*t*) along the *x* and *y* axes

7.3 $$R_{x}(t)=-R(t)\sin[\alpha (t)-\beta (t)]\;\mathrm{and}\;R_{y}(t)=-R(t)\cos[\alpha (t)-\beta (t)],$$

which are given as the sum of the respective translational inertia, the (linear) viscous damping force, and the dead load

7.4 $$R_{x}(t)=-m\ddot{x}(l,t)-c\dot{x}(l,t)\;\mathrm{and}\;R_{y}(t)=-m[g+\ddot{y}(l,t)]-c\dot{y}(l,t),$$

where *c* is the damping coefficient owing to air drag. To model the axial compliance of the system (neglected in the quasi-static calculations), an axial spring of stiffness *k*_c_ is introduced in the clamp, so that the kinematical constraint (2.3) is now replaced by

7.5 $$\begin{array}{rl} x(s,t) & =-\frac{R(t)\cos\beta (t)\cos\alpha (t)}{k_{c}}-\int_{s}\sin[\theta (s,t)+\alpha (t)]\;ds\\ \;\text{and}\;y(s,t) & =-\frac{R(t)\cos\beta (t)\sin\alpha (t)}{k_{c}}-\int_{s}\cos[\theta (s,t)+\alpha (t)]\;ds. \end{array}\}$$

Introducing the reference time $T=\sqrt{ml^{3}/B}$ and the following dimensionless quantities

7.6 $$\begin{array}{rl} \tau & =\frac{t}{T},\;\xi (\tau )=\frac{x(l,t)}{l},\;\eta (\tau )=\frac{y(l,t)}{l}\\ \;\text{and}\;\mu (\tau ) & =\frac{R(t)l^{2}}{B},\;\kappa_{c}=\frac{k_{c}l^{3}}{B},\;\upsilon =c\sqrt{\frac{l^{3}}{mB}},\;\zeta =\frac{gml^{2}}{B}, \end{array}\}$$

a comparison of equations (7.3) with the differential equations (7.4) leads to the following non-dimensionalized differential system, governing the snap-back dynamics

7.7 $$\begin{array}{rl} & \frac{d^{2}\xi (\tau )}{d\tau^{2}}+\upsilon \frac{d\xi (\tau )}{d\tau }=\mu (\tau )\sin[\alpha (\tau )-\beta (\tau )]\\ \;\text{and}\; & \frac{d^{2}\eta (\tau )}{d\tau^{2}}+\upsilon \frac{d\eta (\tau )}{d\tau }+\zeta =\mu (\tau )\cos[\alpha (\tau )-\beta (\tau )]. \end{array}\}$$

Note that the elastica, equation (7.1), is the same as for the quasi-static problem (3.2), but with the two functions of time *R*(*t*) and *β*(*t*) replacing now *P* and *α*, respectively. Therefore, the integration in space for the dynamic problem, equation (7.1), can be computed similarly to the quasi-static case (§3) restricted to the first deformation mode, *n*=1. This integration yields the following relation between the normalized resultant *μ*(*τ*), the rotation of the rod at the free end *θ*(*l*,*τ*), and the resultant inclination *β*(*τ*)

7.8 $$\mu (\tau )={\left[K(h(\tau ))-K(\chi_{0}(\tau ),h(\tau ))\right]}^{2},$$

where

7.9 $$h^{2}(\tau )=\cos^{2}\left(\frac{\theta (l,\tau )+\beta (\tau )}{2}\right),\;\chi_{0}(\tau )=-\arcsin\left[\frac{1}{h(\tau )}\cos\left(\frac{\beta (\tau )}{2}\right)\right].$$

On the other hand, the normalized displacements at the free end, *ξ*(*τ*) and *η*(*τ*), follow from the displacement field for the quasi-static problem, equation (4.1), and from the axial compliance of the clamp, equation (7.5), as

7.10 $$\begin{array}{rl} \xi (\tau ) & =-\frac{\mu (\tau )\cos\beta (\tau )\cos\alpha (\tau )}{\kappa_{c}}\\ & \;-\frac{2\;h(\tau )}{\sqrt{\mu (\tau )}}\left\{\text{Cn}\left[\sqrt{\mu (\tau )}+K(\chi_{0}(\tau ),h(\tau )),h(\tau )\right]-\text{Cn}[K(\chi_{0}(\tau ),h(\tau )),h(\tau )]\right\}\\ \;\text{and}\;\eta (\tau ) & =-1-\frac{\mu (\tau )\cos\beta (\tau )\sin\alpha (\tau )}{\kappa_{c}}\\ & \;+\frac{2}{\sqrt{\mu (\tau )}}\left\{E\left[\text{am}\left(\sqrt{\mu (\tau )}+K(\chi_{0}(\tau ),h(\tau )),h(\tau )\right)\right]-E(\chi_{0}(\tau ),h(\tau ))\right\}. \end{array}\}$$

The substitution of the expressions (7.8) and (7.10) for *μ*(*τ*), *ξ*(*τ*), *η*(*τ*) in the differential equations (7.7) leads to a nonlinear second-order differential implicit system for the evolution of the free end rotation *θ*(*l*,*τ*) and for the resultant inclination *β*(*τ*). For given initial conditions and rotation law *α*(*τ*), the integration of the differential system (7.7) can finally be numerically performed.

In the present analysis, initial rest condition for the system and a linear time evolution law for the clamp rotation are considered, so that

7.11 $$\alpha (t)=\alpha_{0}+\omega t,$$

where *α*_0_ is the initial clamp rotation and *ω* is the clamp angular velocity, and which can be rewritten in terms of dimensionless time as

7.12 $$\alpha (\tau )=\alpha_{0}+\Omega \tau ,$$

with *Ω* being the clamp angular velocity referred to the dimensionless time, *Ω*=*ωT*. To avoid a trivial solution, the evolution of the system was analysed starting at *τ*=0 from an ‘almost’ undeformed state for the rod, namely the initial clamp inclination was set to be *α*_0_=10^−5^. The rest condition for the system at *τ*=0 implies that initially the inclination *β*(0) coincides with the clamp inclination *α*(0), so that the force resultant *R*(0) momentarily coincides with the dead load *P*, and that the lumped mass has null velocity,

7.13 $$\beta (0)=\alpha (0),\;\mu (0)=\zeta \;\mathrm{and}\;\dot{\xi }(0)=\dot{\eta }(0)=0.$$

The four conditions (7.13) imply four initial conditions for the resultant inclination *β*(*τ*) and the free end rotation *θ*(*l*,*τ*)

7.14 $$\beta (0)=\alpha_{0},\;\theta (l,0)=\theta_{l0},\;\dot{\beta }(0)={\dot{\beta }}_{0}\;\mathrm{and}\;\dot{\theta }(l,0)={\dot{\theta }}_{l0},$$

where the values of *θ*_*l*0_, ${\dot{\beta }}_{0}$ and ${\dot{\theta }}_{l0}$ can be computed using equations (7.8) and (7.10).

The evolution of the resultant inclination *β*(*τ*) and the free end rotation *θ*(*l*,*τ*) are obtained from the numerical integration of the nonlinear differential implicit equations (7.7) through the function NDSolve in Mathematica^©^ (v. 10). Considering the clamp angular velocity *ω*=0.014 rad s^−1^ and assuming a small normalized damping coefficient *υ*=0.069 and high normalized axial stiffness *κ*_c_=310.647, the numerical integration has been performed at varying the normalized mass parameter, namely for *ζ*={1.157,1.987,2.818,3.648,4.478} corresponding to *P*/*P*_cr_={0.469,0.805,1.142,1.479,1.815} and which are representative of some of the experimental set-ups considered in §8.

The massless model is able to capture reasonably well the dynamics of the system for values of the normalized mass parameter *ζ* lower than 5, corresponding to *P*/*P*_cr_<2. For higher values of *ζ*, the numerical integration fails to converge soon after the snap-back instability occurs, namely, the free end experiences a very fast and large oscillation in its acceleration, and the Mathematica solver reveals that the differential system becomes stiff. The encountered difficulty in the numerical treatment is related to the fact that rod configuration is imposed to assume the first quasi-static mode during the dynamics, a condition which becomes not representative at values *P*/*P*_cr_>2. Indeed, it is experimentally shown in §8 that for this loading condition the dynamics of the rod is characterized by both transverse and longitudinal oscillation, whereas the present simplified model is reliable only when transverse oscillations prevail in the dynamic response.

### (b) Finite-element analysis

A finite-element analysis of the system was performed in Abaqus with the purpose to provide a full description of the dynamics of the rod for every loading condition, thus overcoming the limitations found with the simplified model. The rod is modelled through 100 linear elastic planar beam elements, where the first element has the external edge constrained by the rotating clamp while the lumped mass *m* is attached on the external edge of the final element. Introducing energy dissipation through Rayleigh damping, large displacement analyses were performed through the following two steps:

Step 1. Static—the gravitational force is applied in order to establish the deformed initial configuration. Here a quasi-static deformation is produced by the weight of both the elastic rod and the lumped mass (although the effect of the former is much smaller than the effect induced by the latter);
Step 2. Dynamic implicit—a slow rotation to the clamped end is imposed as a boundary condition. The analysis takes into account the inertial forces generated during the clamp rotation.

Simulations were run considering geometry, material, inertia and clamp angular velocity representative of the experimental set-up described in the next section. Rayleigh damping was set through the mass-proportional damping coefficient *A*=10^−2^ *s*^−1^ and the stiffness-proportional damping coefficient *B*=5×10^−3^ *s*. Several low rotation velocities for the clamp have been investigated (*ω*={0.014,0.06,0.14} rad s^−1^), basically showing no influence of small *ω* on the dynamics of the system.

## 8. Experiments versus modelling for the elastica compass and elastica catapult

**Figure 13.**
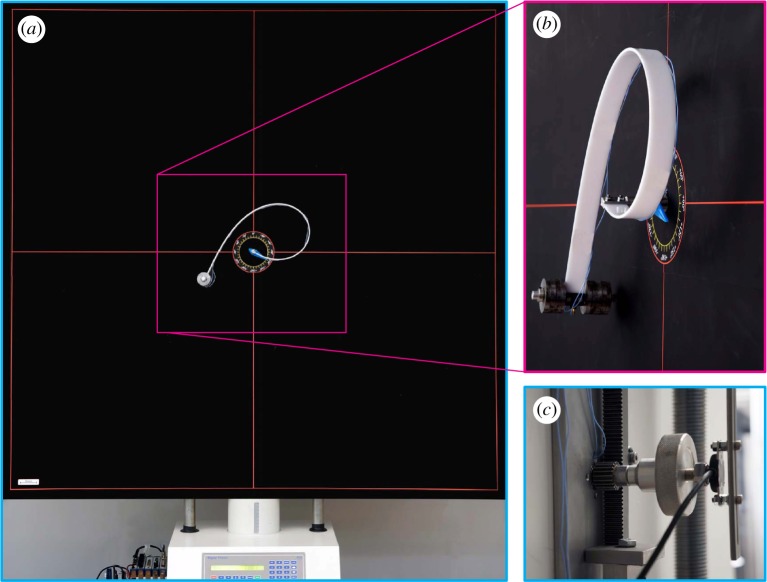
Experimental set-up for the elastica compass and the elastica catapult. (*a*) Global view of the experimental set-up, (*b*) detail of the elastic rod and (*c*) rear view detail of the rack and pinion mechanism to impose clamp rotation and from which the rotary position sensor is visible. (Online version in colour.)

An experimental set-up was designed and manufactured at the Instabilities Lab of the University of Trento (http://ssmg.unitn.it) for the analysis of the rotating clamped elastic rod (figure 13). A rack and pinion actuator (figure 13*c*) was used to transform the linear motion of an electromechanical testing machine (ELE Tritest 50 from ELE International Ltd) into the rotation of the clamp at required angular speeds (different low clamp velocities were tested in the experiments, without noting substantial differences, so that only results for *ω*=0.014 rad s^−1^ are reported). The angular position of the rotating clamp was measured through a contactless rotary position sensor (NRH280DP/180/360). The moment transmitted to the clamp was measured with a lever system connected to a load cell (Leane DBBSN, RC 10 kg), as evident from figure 13*c*.

During the tests, the modulus of the acceleration of the lumped mass, attached at the end of the beam

$$a(l,t)=\sqrt{\ddot{x}{\left(l,t\right)}^{2}+\ddot{y}{\left(l,t\right)}^{2}}$$

was obtained mounting two miniaturized mono-axial accelerometers (352A24, PCB piezotronics, 0.8*g*) perpendicular to each other. All the data were acquired with a NI compactRIO system interfaced with Labview 2013 (from National Instruments).

Two rods were used, both made up of a solid polycarbonate strip (white 2099 Makrolon UV from Bayer, *E*=2350 MPa, Poisson’s ratio *ν*=0.37 and volumetric mass density *ρ*=1180 kg m^−3^). One rod, 345 mm long, has a 25×3 mm cross section and the other, 695 mm long, has a 25×2.85 mm cross section. The masses attached at the end of the two rods were chosen to produce the following values of *P*/*P*_cr_={0.469,0.805,1.142,1.479,1.815,3.813,5.405,6.998,7.795}.

**Figure 14.**
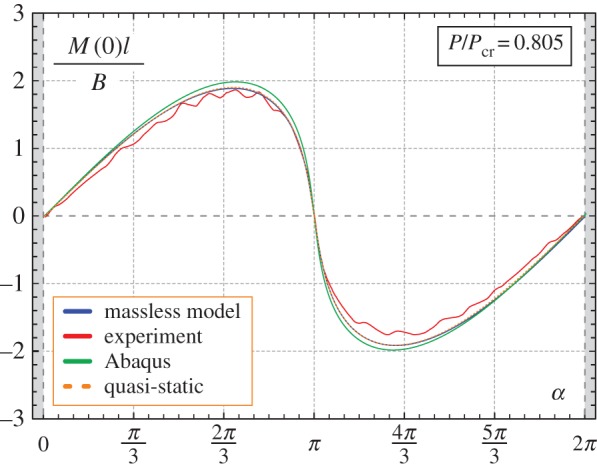
Comparison between the theoretically predicted and experimentally measured values of the moment at the clamp *M*(0) at varying the clamp angle *α* for the elastica compass, in the case *P*=0.805*P*_cr_.(Online version in colour.)

**Figure 15.**
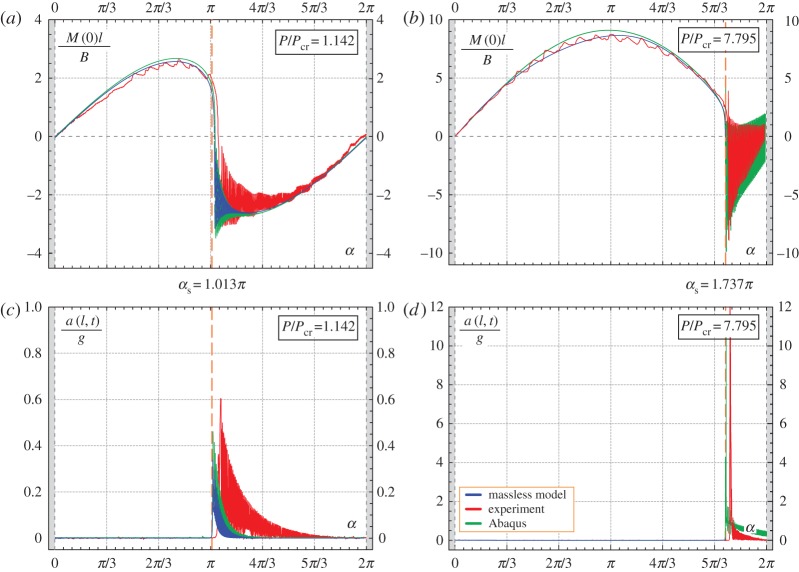
Comparison between the theoretically predicted and experimentally measured values of the moment at the clamp *M*(0) (*a,b*) and of the total acceleration of the lumped mass $a(l,t)=\sqrt{\ddot{x}{\left(l,t\right)}^{2}+\ddot{y}{\left(l,t\right)}^{2}}$ (*c*,*d*) at varying the clamp angle *α* for the elastica catapult, in the cases *P*=1.142*P*_cr_ (left column) and *P*=7.795*P*_cr_ (right column). The curves highlight the presence of snap-back dynamics during the clamp rotation when the angle *α*_s_ is approached. In particular, a change in sign for the moment at the clamp *M*(0) is observed as well as high acceleration of the rod’s end after the snap-back phenomena. (Online version in colour.)

**Figure 16.**
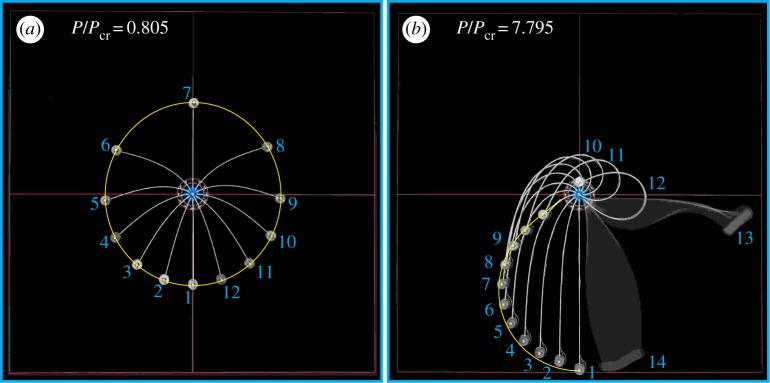
Superimposed photos at different clamp angles *α* for (*a*) the elastica compass, *P*/*P*_cr_=0.805, and (*b*) the elastica catapult, *P*/*P*_cr_=7.795. The blurred images in the photo on the right were taken during the post-snap dynamics at 1/20 s shutter speed. The theoretical trajectory of the ‘pencil lead’, obtained from the quasi-static analysis, is reported as continuous yellow line. (Online version in colour.)

Results of experiments are shown in figures 3, 14, 15 and 16. In particular, in figure 3 the experimental points corresponding to the snap angles have been recorded from a change in sign of the moment measured at the clamp. The same moment *M*(0) is reported in figures 14 and 15 as a function of the clamp rotation *α* for the case of the elastica compass (*P*/*P*_cr_=0.805, where a quasi-static path is followed, figure 14) and in the case of the elastica catapult (*P*/*P*_cr_=1.142 and *P*/*P*_cr_=7.795, where the dynamics prevails after snap-back, figure 15*a,b*, respectively). In the case of the elastica compass the acceleration *a*(*l*,*t*) was found to be negligible (the maximum value was about 10^−2^ *g*), whereas in the case of the elastica catapult the acceleration *a*(*l*,*t*) (reported in figure 15*c*,*d*) was found to have a peak of about 0.6*g* and 55*g* for *P*/*P*_cr_=1.142 and *P*/*P*_cr_=7.795, respectively. These peaks in the acceleration were found to occur just after the snap-back instability.

Photos taken at different rotation angles are superimposed in figure 16 for two cases in which the system behaves as an elastica compass (figure 16*a*, *P*/*P*_cr_=0.805) and an elastica catapult (figure 16*b*, *P*/*P*_cr_=7.795). In the latter case, the photo labelled 12 corresponds to the last photo taken before snap-back instability (*α*=1.744*π*), whereas photos 13 and 14 are blurred because they were taken (using a NEX-5N Sony camera) during post-snap dynamics. Note that the shutter speed was set on 1/20 s, which provides a measure of the velocity of the rod during snap-back. In figure 16, the position of the attached lumped mass is compared with the theoretically predicted rod’s end trajectory (obtained from the quasi-static analysis and reported as yellow line), showing a very nice agreement.

Movies of the experiments, taken by both high-speed camera Sony PXW-FS5K (240 fps) and a Sony Handycam HDR-XR550, are available as electronic supplementary material.

## 9. Conclusion

In the simplest set-up for a flexible robot arm, an elastic rod is subject to a prescribed slow rotation at one end and to a concentrated mass in a gravity field at the other. Solving this system through the elastica has evidenced a discriminating role for the applied load. When the load is smaller than the buckling load (for the straight configuration), the loaded end describes a closed continuous curve (not far from an ellipse), whereas when it is higher the continuous displacement of the loaded end terminates in an unstable geometrical form, from which a snap-back instability leads to a non-adjacent configuration. In the former case, the system behaves as an ‘elastica compass’ (so that the loaded end of the arm would describe a circle if the rod would be rigid), whereas in the latter case, the system behaves as an ‘elastica catapult’ (so that the mass would be thrown away if detachment were allowed). The dynamic motion after the snap instability has been analysed with a standard finite-element program and an *ad hoc* developed software. The quasi-static and dynamical behaviours have been validated with a specifically designed experimental set-up. Results show that the basic problem of soft robot arm addressed in this article, and systematically explored in terms of mechanical performances, can be analytically and numerically described with a great accuracy and therefore is ready for exploitation in real devices.

## Footnotes

1 The ancient catapults derived their launching power either from gravity (in the case of the so-called trebuchet) or from the elastic energy stored in a coil of twisted sinew, concentrated at the pivot point of the arm (in the case of the so-called onager catapult). Leonardo da Vinci has sketched a series of catapults (collected in the Codex Atlanticus) where the elastic energy for propulsion was stored through flexure of the catapult arm, a mechanism similar to that treated in this article.

## Data accessibility

Calculations were performed using Wolfram’s MATHEMATICA v. 10. Experimental data are shown in figures 3, 13–16 and in the electronic supplementary material.
